# The New Medical Bill

**Published:** 1880-07

**Authors:** 


### THE NEW MEDICAL BILL.

By the request of many of our subscribers we publish the full text of the act recently passed by the Legislature, regulating the licensing of physicians and surgeons in this State. It will be seen that the last clause of Section 6 renders the bill in some respects nugatory. We are informed that it was tacked on to the original bill in the Senate, through the influence of the Senator from Erie County. It is in the interest of unqualified practitioners and quacks now practicing in this State.

The clause in § 4 requiring all applicants to pay a fee of $20 for the examination and endorsement of their diploma is discreditable to the medical profession of New York. A graduate of Harvard, Jefferson, or any other college outside of the State is thus compelled to pay a fee of $20 to some, perhaps insignificant medical school in order that his diploma may be recognized as giving him a right to practice medicine in this State. In such cases at least the fee is exorbitant, and is of benefit not to the profession, but to the colleges. A smaller fee would also remove the temptation which some faculties may have, of desiring to pass as many applicants as possible, thus facilitating the introduction of unqualified men into this State. We fear that the bill as passed will not be of much benefit to the profession.

AN ACT ENTITLED "AN ACT TO REGULATE THE LICENSING OF PHYSICIANS AND SURGEONS." PASSED MAY 29, 1880; THREE-FIFTHS BEING PRESENT.

*The People of the State of New York, represented in Senate and Assembly, do enact as follows:*

SECTION 1. A person shall not practice physic or surgery within the State unless he is twenty one years of age, and either has been heretofore authorized so to do, pursuant to the laws in force at the time of his authorization, or is hereafter authorized so to do as prescribed by chapter seven hundred and forty-six of the laws of eighteen hundred and seventy-two, or by subsequent sections of this act

§ 2 Every person now lawfully engaged in the practice of physic and surgery within the State shall, on or before the first day of October, eighteen hundred and eighty, and every person hereafter duly authorized to practice physic and surgery shall, before commencing to practice, register in the Clerk's Office of the County where he is practicing, or intends to commence the practice of physic and surgery, in a book to be kept by said clerk, his name, residence and place of birth, together with his authority for so practicing physic and surgery as prescribed in this act. The person so registering shall subscribe and verify by oath or affirmation, before a person duly qualified to administer oaths under the laws of the State, an affidavit containing such facts, and whether such authority is by diploma or license, and the date of the same and by whom granted, which, if willfully false, shall subject the affiant to conviction and punishment for perjury The County Clerk to receive a fee of twenty-five cents for such registration, to be paid by the person so registering.

§ 3 A person who violates either of the two preceding sections of this act, or who shall practice physic or surgery under cover of a diploma illegally obtained, shall be deemed to be guilty of a misdemeanor, and on conviction shall be punished by a fine of not less than fifty dollars nor more than two hundred dollars for the first offense, and for each subsequent offense by a fine of not less than one hundred dollars nor more than five hundred dollars, or by imprisonment for not less than thirty days nor more than ninety days, or both. The fine when collected shall be paid, the one half to the person or corporation making the complaint, the other half into the county treasury.

§ 4. A person coming to the state from without the state may be licensed to practice physic and surgery, or either, within the state, in the following manner: If he has a diploma conferring upon him the degree of doctor of medicine, issued by an incorporated university, medical college, or medical school without the state, he shall exhibit the same to the faculty of some incorporated medical college or medical school of this state, with satisfactory evidence of his good moral character, and such other evidence, if any, of his qualifications as a physiciâns or surgeon, as said faculty may require. If his diploma and qualifications are approved by them, then they shall indorse said diploma, which shall make it for the purpose of his license to practice medicine and surgery within this state the same as if issued by them. The applicant shall pay to the dean of said faculty the sum of twenty dollars for such examination and indorsement. This indorsed diploma shall authorize him to practice physic and surgery within the state upon his complying with the provisions of section two of this act.

§ 5. The degree of doctor of medicine, lawfully conferred by any incorporated medical college or university in this state, shall be a license to practice physic and surgery within the state after the person to whom it is granted shall have complied with section two of this act.

§ 6. Nothing in this act shall apply to commissioned medical officers of the United States army or navy, or of the United States marine hospital service Nor shall it apply to any person who has practiced medicine and surgery for ten years last past, and who is now pursuing the study of medicine and surgery in any legally incorporated medical college within this state, and who shall graduate from and receive a diploma wlthin two years from the passage of this act.

§ 7. All acts, or parts of acts inconsistent with the provisions of this act are hereby repealed.

Office of the Secretary of State. } ss.<br>

STATE OF NEW YORK, }

I have compared the preceding with the original law on file in this office, and do hereby certify that the same is a correct transcript therefrom and of the whole of said original law. JOSEPH B. CARR,

Secretary of State.

